# Clinical characteristics, risk factors and outcomes of *Klebsiella pneumoniae* pneumonia developing secondary *Klebsiella pneumoniae* bloodstream infection

**DOI:** 10.1186/s12890-023-02394-8

**Published:** 2023-03-28

**Authors:** Juan Chen, Jiahui Li, Fangfang Huang, Junjie Fang, Yang Cao, Kai Zhang, Hongwei Zhou, Jiachang Cai, Wei Cui, Chensong Chen, Gensheng Zhang

**Affiliations:** 1 Department of Critical Care Medicine, Second Affiliated Hospital, Zhejiang University School of Medicine, 88 Jiefang Road, Hangzhou, 310009 China; 2 Department of Intensive Care Unit, Ningbo Fourth Hospital, 291 Donggu Road, Dandong Street, Ningbo, 315700 Zhejiang China; 3 Department of Critical Care Medicine, Yuyao People’s Hospital, Yuyao, 315400 China; 4 Department of Clinical Microbiology Laboratory, Second Affiliated Hospital, Zhejiang University School of Medicine, Hangzhou, 310009 China; 5 Zhejiang Province Clinical Research Center for Emergency and Critical Care Medicine, 88 Jiefang Road, Hangzhou, 310009 China

**Keywords:** Klebsiella pneumoniae, KP pneumonia, KP pneumonia developing secondary KP bloodstream infection, Clinical characteristics, Risk factors, Outcomes

## Abstract

**Purpose:**

The clinical characteristics of *Klebsiella pneumoniae* (KP) pneumonia and KP bloodstream infection (KP-BSI) are often reported, while the risk factors for KP pneumonia developing into secondary KP-BSI (KP-pneumonia/KP-BSI) are largely unknown. Therefore, this study attempted to investigate the clinical characteristics, risk factors and outcomes of KP-pneumonia/KP-BSI.

**Methods:**

A retrospective observational study was conducted at a tertiary hospital between January 1, 2018, and December 31, 2020. The patients were divided into groups of KP pneumonia alone and KP pneumonia/KP-BSI, and the clinical information were collected from medical records electronic system.

**Results:**

A total of 409 patients were finally recruited. According to the multivariate logistic regression analysis, male sex (adjusted odds ratio [aOR] 3.7; 95% CI, 1.44–9.5), immunosuppression (aOR, 13.52; 95% CI, 2.53,72.22), APACHE II score higher than 21 (aOR, 3.39; 95% CI, 1.41–8.12), serum procalcitonin (PCT) levels above 1.8 ng/ml (aOR, 6.37; 95% CI, 2.67–15.27), ICU stay of more than 2.5 days before pneumonia onset (aOR, 1.09; 95% CI, 1.02,1.17), mechanical ventilation (aOR, 4.96; 95% CI, 1.2,20.5), Klebsiella pneumoniae isolates producing extended spectrum β-lactamase (ESBL-positive KP) (aOR, 12.93; 95% CI, 5.26–31.76), and inappropriate antibacterial therapy (aOR, 12.38; 95% CI, 5.36–28.58) were independent factors of KP pneumonia/KP BSI. In comparison with the patients with KP pneumonia alone, the patients with KP pneumonia/KP BSI showed an almost 3 times higher incidence of septic shock (64.4% vs. 20.1%, *p* < 0.01), a longer duration of mechanical ventilation, and longer lengths of ICU stay and total hospital stay (median days, 15 vs. 4,19 vs. 6, 34 vs. 17, respectively, both *p* < 0.01). Additionally, the overall in-hospital crude mortality rate in the patients with KP-pneumonia/KP-BSI was more than two times higher than that in those with KP pneumonia alone (61.5% vs. 27.4%, *p* < 0.01).

**Conclusion:**

Factors including male sex, immunosuppression, APACHE II score higher than 21, serum PCT levels above 1.8 ng/ml, ICU stay of more than 2.5 days before pneumonia onset, mechanical ventilation, ESBL-positive KP, and inappropriate antibacterial therapy are independent risk factors for KP pneumonia/KP-BSI. Of note, the outcomes in patients with KP pneumonia worsen once they develop secondary KP-BSI, which merits more attention.

## Background

Klebsiella pneumoniae (KP) is a gram-negative, encapsulated, bacterium in the environment [1]. The bacterium typically colonizes human mucosal surfaces, including the gastrointestinal (GI) tract and oropharynx [2]. Once the bacteria enter the body, they can exhibit a high degree of toxicity and antibiotic resistance [3]. KP pneumonia accounts for approximately 11.8% of all hospital-acquired pneumonia cases cases worldwide and nearly 15% of all cases of pneumonia in developing countries [3]. Patients with KP pneumonia often display a poor prognosis. Even with optimal treatment, the mortality rate of KP pneumonia is between 30 and 50% [4, 5]. KP bloodstream infection (KP-BSI) is one of the most fatal infections [6, 7] and is associated with high mortality ranging from 20 to 40% [8], whereas mortality was reported to reach 67.6% in ICU patients [9]. The most important prognostic variables in KP-BSI are the main site of infection (i.e., pneumonia more than urinary tract infection), the severity of the underlying disease (i.e., septic shock or a higher APACHE II score) and the adequacy of antimicrobial therapy. Indeed, the prognosis, including hospital stays, mortality and hospitalization costs, is usually worse in patients with KP pneumonia once they are complicated with bacteremia [10]. However, whether the outcomes of patients with KP pneumonia developing into secondary KP-BSI (KP-pneumonia/KP-BSI) would be worse than in the patients with KP-pneumonia alone or which risk factors contribute to the development of secondary KP-BSI from KP pneumonia alone are largely unknown.

Although many studies have focused on KP-BSI [8, 11–18], there are some limitations, which are as follows: (1) Many studies have demonstrated that several comorbidities are risk factors for KP-bacteremia, including diabetes, cancer, chronic liver disease, and biliary tract disease [8, 11, 14, 15, 17, 18]. However, these risk factors were inconsistent, possibly because of different primary infections, such as KP-caused liver abscess [17], urinary tract infection [18] or biliary tract infection/intraperitoneal sources [8]. At present, the risk factors for KP-pneumonia/KP-BSI remain unclear. (2) Some studies have suggested that pneumonia as a primary infection source might be an important prognostic factor for crude 28-day mortality of KP-BSI [12, 13]. However, these studies focused primarily on the prognosis of secondary KP-BSI but less on the direct comparison of the prognoses between primary KP pneumonia alone and KP-pneumonia/KP-BSI. (3) Although a recent single-center study investigated the independent risk factors for developing secondary KP-BSI from underlying pneumonia [16], the study only included patients infected with carbapenem-resistant KP (CRKP) bacteria. In addition, the study recruited very specific ICU patients and had a relatively small sample size (*n* = 76) [16]. Therefore, the clinical features of KP-pneumonia/KP-BSI are still largely unknown.

Given the poor prognosis of KP-pneumonia/KP-BSI and the fact that the risk factors for KP- pneumonia to develop secondary KP-BSI have not been investigated, it is urgent and critical to identify some preventable factors to inhibit the development of secondary KP-BSI in patients with KP alone. Thus, we performed this study to analyze the characteristics, risk factors and prognoses of patients with KP-pneumonia/KP-BSI compared with patients who had KP-pneumonia alone.

## Materials and methods

### Patients and study design

A retrospective cohort study was conducted between January 2018 and December 2020 at the Second Hospital affiliated with Zhejiang University School of Medicine, a 3,200-bed tertiary facility in Hangzhou, China. This study got approval from the Ethics Committee of the Second Hospital affiliated with Zhejiang University School of Medicine (No 2021–0674). Due to the retrospective analysis, the Ethics Committee decided to waive the need for informed consent of patients.

All patients admitted to our hospital with positive KP cultures from sputum or BALF were recruited. The exclusion criteria were as follows: a) KP was considered colonizing or contaminating bacteria [19]; b) patients with polymicrobial respiratory infections; c) patient data were incomplete or missing; d) age < 18 years; e) if KP was found in multiple respiratory cultures in the same patient, these patients were included only once when the first positive lower respiratory tract specimen was obtained. and f) Patients with KP pneumonia and KP-BSI simultaneously on admission, which made it difficult to differentiate primary or secondary KP-BSI.

### Definitions

The diagnoses of pneumonia, hospital-acquired pneumonia (HAP), ventilator-associated pneumonia (VAP) and community-acquired pneumonia (CAP) were based on the CDC definition [20, 21]. KP bacteremia refers to the detection of KP in blood culture specimens. KP bacteremia is defined as at least one positive blood culture, as well as clinical features consistent with systemic inflammatory response syndrome [22]. KP-pneumonia/KP-BSI was diagnosed based on the isolation of KP from blood culture specimens from patients with KP pneumonia, and when other sources of infection and specimen contamination were excluded, as stated in Bloodstream Infection Events [23]. Criteria for performance of ESBL-K. pneumoniae test are performed by Broth microdilution or Disk diffusion clavulanate inhibition tests according to CLSI standards [24]. ESBL-K. pneumoniae positive criteria: A ≥ threefold concentration decrease in an MIC for either antimicrobial agent tested in combination with clavulanate vs the MIC of the agent when tested alone = ESBL (eg, ceftazidime MIC = 8 μg/mL; ceftazidime-clavulanate MIC = 1 μg/mL). Immunosuppression includes chemotherapy or radiotherapy within 30 days before admission, solid organ transplantation or hematopoietic stem cell transplantation, and corticosteroid therapy with a daily dose of ≥ 25 mg lasting more than 1 month or a cumulative dose of 700 mg for > 3 months [25]. Septic shock was defined by the new definition of sepsis-3 [26]. Appropriate antibiotic therapy was considered if at least one antibiotic preparation was consistent with in vitro susceptibility results [27, 28].

### Data collection

Patient data were gathered by reviewing electronic medical records. The demographic data, including age and sex, APACHE II score within 24 h of KP pneumonia onset, Charlson Comorbidity Index (CCI) score, sequential organ failure assessment (SOFA) score, underlying diseases, nosocomial infection, previous treatments (such as surgery, blood transfusion, mechanical ventilation, renal replacement therapy, immunosuppressive agents, parenteral nutrition, chemotherapeutic agents, radiation therapy), and outcomes, including the duration of mechanical ventilation, the length of ICU stay or total hospital stay, occurrence of septic shock and in-hospital mortality, were also recorded. In addition, microbiological data, such as bacterial sensitivity to antibiotics, were also gathered.

### Species identification and antibiotic sensitivity test

In the microbiology laboratory, sputum and BALF samples were cultured by using a Colombian agar plate containing 5% sheep blood (Thermo Fisher Scientific, USA), while blood samples were grown using the BacT/Alert 3D system (bioMérieux, Marcyl’Etoile, France). Species identification was performed using Bruker Daltonics data analysis. The antibiotic sensitivity test adopted the VITEK 2 (Card No.: ast-gn16; ast-gp67) system recommended by the Clinical and Laboratory Standards Institute (CLSI) or the Kirby Bauer disk diffusion method (Oxoid, UK).

### Statistical analysis

SPSS 25.0 software (IBM Corp, Armonk, NY, USA) was used for statistical analysis. A two-tailed *p* < 0.05 was considered statistically significant. The analysis of continuous variables is presented as the mean and standard deviation if normally distributed and as the median and interquartile range (IQR) if not normally distributed. The analysis of continuous variables was conducted using Student’s t test or the Mann–Whitney U test, while Pearson χ2 or Fisher’s exact test was used for analyzing classified variables. A stepwise logistic regression multivariable model was built using variables with significance at *p* < 0.05 in the univariate analysis.

## Results

### Demographic and clinical characteristics

**Fig. 1:**
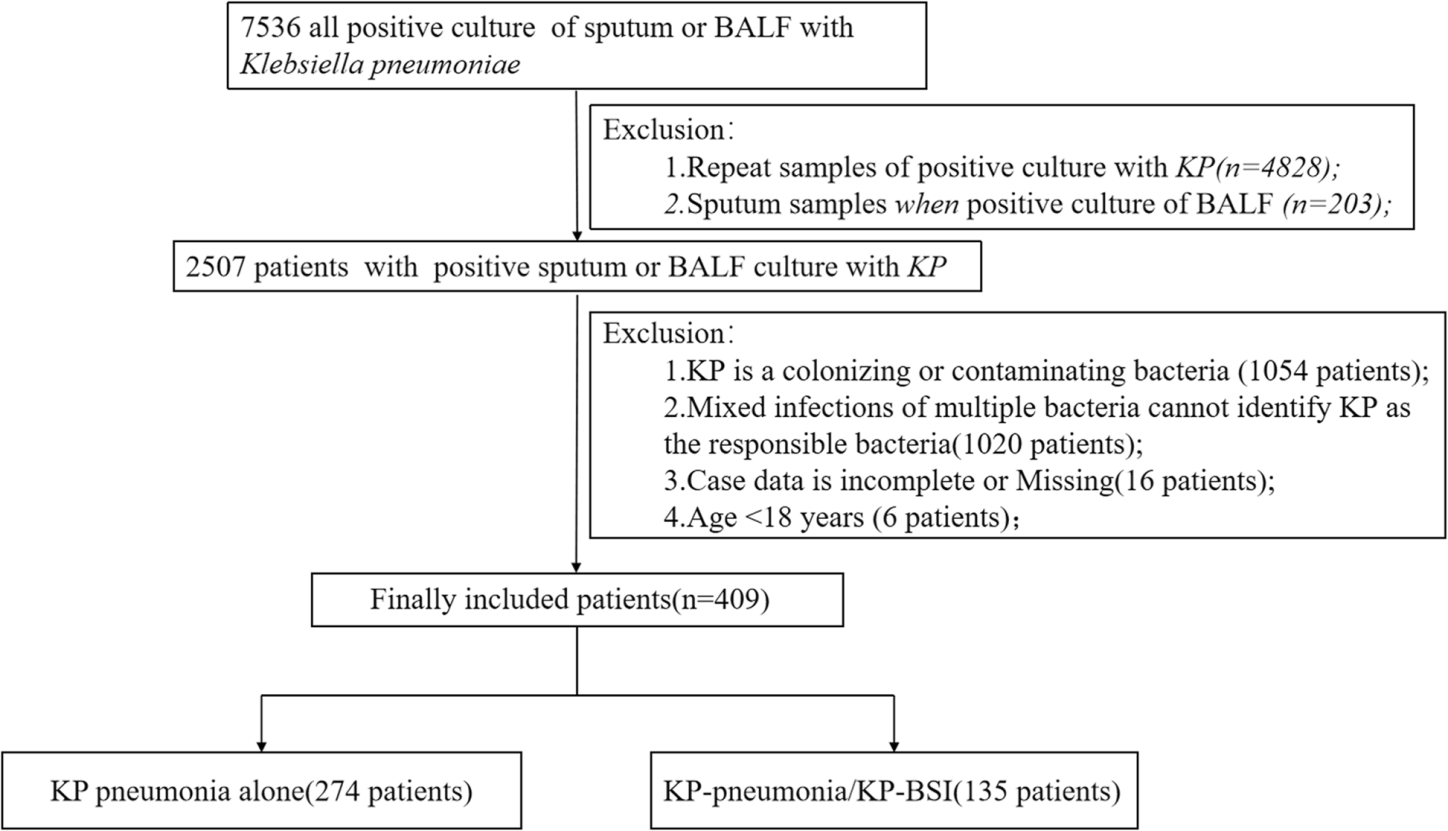
Flowchart of study participant enrollment. Abbreviations: BALF, broncho alveolar lavage fluid; KP, Klebsiella pneumoniae; KP-BSI, KP pneumonia secondary KP bloodstream infections

A total of 7536 lower respiratory tract culture specimens containing KP were primarily included, and 409 patients were finally recruited, including 274 patients with KP pneumonia alone (67.0%) and 135 patients with KP pneumonia/KP-BSI (33.0%) (Fig. 1).

The demographic and clinical characteristics of these patients are summarized in Table 1. The median age was 65 years (IQR, 54.5–75.0), and 73.6% (301/409) were male. In terms of sex, there were more males in the KP-pneumonia/KP-BSI group than in the KP-pneumonia alone group (80.1% vs. 70.5%, *p* < 0.05). Cerebrovascular accident or traumatic brain injury (both 54.5%) was the most common comorbidity, followed by diabetes (19.6%) and chronic cardiac insufficiency (14.7%). A significantly high proportion of patients with chronic cardiac insufficiency, chronic renal failure or immunosuppression was observed in the group of patients with KP-pneumonia/KP-BSI (all *p* < 0.05).

**Table 1:** Baseline characteristics of patients with KP pneumonia alone and KP-pneumonia/KP-BSI

Characteristics	Total (*n* = 409)	KP pneumonia alone (*n* = 274)	KP-pneumonia/KP-BSI (*n* = 135)	*P*-value
Age, median years (IQR)	65 (54.5, 75)	65 (56, 75)	64 (51, 76)	*P* = 0.39
Male sex	301 (73.6%)	193 (70.4%)	109 (80.1%)	*P* = 0.04
Co-morbidities (n, %)				
Diabetes mellitus	80 (19.6%)	52 (18.9%)	28 (20.7%)	*P* = 0.07
Chronic cardiac insufficiency	60 (14.7%)	32 (11.7%)	28 (20.7%)	*P* = 0.02
COPD or Severe asthma	43 (10.5%)	31 (11.3%)	12 (8.9%)	*P* = 0.45
Chronic renal failure	16 (3.9%)	7 (2.6%)	9 (6.7%)	*P* = 0.05
Cerebrovascular disease	224 (54.5%)	159 (57.8%)	65 (47.8%)	*P* = 0.06
Trauma	67 (16.4%)	40 (14.6%)	27 (20%)	*P* = 0.17
Immunosuppressive	28 (6.8%)	12 (4.4%)	16 (11.9%)	*P* = 0.01
Solid tumor	44 (10.8%)	33 (12%)	11 (8.1%)	*P* = 0.23
Previous treatment				
Blood transfusion	88 (21.5%)	31 (11.3%)	57 (42.2%)	*P* < 0.01
Mechanical ventilation	299 (73.1%)	175 (63.9%)	124 (91.9%)	*P* < 0.01
Surgery	220 (53.8%)	148 (54%)	72 (53.3%)	*P* = 0.90
Parenteral nutrition	85 (20.8%)	48 (17.5%)	37 (27.4%)	*P* = 0.02
Renal replacement therapy	49 (12%)	17 (6.2%)	32 (23.7%)	*P* < 0.01
Invasive devices				
Indwelling urinary catheter	329 (80.6%)	205 (74.8%)	124 (92.5%)	*P* < 0.01
central line catheter	300 (73.3%)	180 (65.7%)	120 (88.9%)	*P* < 0.01
Intraperitoneal drainage	23 (5.6%)	15 (5.5%)	8 (5.9%)	*P* = 0.85
SOFA score, median (IQR)	6 (4,9)	5 (3,8)	8.5 (5,13)	*P* < 0.01
APACHE II score, median (IQR)	19 (13,25)	17 (12,22)	24.5 (18,30)	*P* < 0.01
ACCI, median (IQR)	3 (2,5)	3 (2,5)	3 (1.5,5)	*P* = 0.60
Pitt Bacteremia Score, median (IQR)	5 (2,7)	4 (2,6)	1 (0,3)	*P* < 0.01
Hospitalization ward (n, %)				
ICU stay	273 (66.4%)	159 (57.8%)	114 (83.8%)	*P* < 0.01
Prior antibiotic use time	1 (0,6)	0 (0,3)	5 (1,15)	*P* < 0.01
Prior hospital stay, median days (IQR)	3 (1,11)	2 (1,7)	7 (1,17.5)	*P* < 0.01
Prior ICU stay, median days (IQR)	1 (0,4)	0 (0,2)	3 (0,8)	*P* < 0.01
Prior Mechanical ventilation, median days (IQR)	2 (0,5)	3 (0,4)	2 (0,7)	*P* = 0.06
Nosocomial infection (n, %)	269 (65.5%)	150 (54.5%)	119 (87.5%)	*P* < 0.01
ESBL-positive KP	196 (47.9%)	81 (29.6%)	115 (85.2%)	*P* < 0.01
Prior hospitalized^a^	144 (35.2%)	81 (29.6%)	63 (46.7%)	*P* < 0.01
Prior mechanical ventilation^a^	68 (16.6%)	31 (11.3%)	37 (27.4%)	*P* < 0.01
Prior antibiotic exposure^a^	141 (34.5%)	80 (29.2%)	61 (45.2%)	*P* < 0.01

*Abbreviations*: *IQR* Interquartile range, *COPD* Chronic obstructive pulmonary disorder, *ACCI* Age-adjusted Charlson Comorbidity Index, *SOFA* Sequential organ failure assessment, *APACHE* Acute physiology and chronic health evaluation, *ICU* Intensive care unit, *ESBL-positive KP* klebsiella pneumoniae isolates producing extended spectrum β-lactamase, *KP* Klebsiella pneumoniae, *KP pneumonia /KP-BSI* KP pneumonia secondary Klebsiella pneumoniae bloodstream infections

^a^in recent 3 months

Compared with the patients with KP pneumonia alone, the group of patients with KP-pneumonia/KP-BSI had a greater percentage of patients who had renal replacement therapy (23.7% vs. 6.2%, *p* < 0.01) and parenteral nutrition (27.4% vs. 17.5%, *p* < 0.01) and had a greater need for blood transfusion (42.2% vs. 11.3%, *p* < 0.05). The ratio of patients requiring central venous catheters or indwelling urinary catheters was also significantly increased (88.9% vs. 65.7%; 92.5% vs. 74.8%, respectively, both *p* < 0.01). Additionally, the patients with KP-pneumonia/KP-BSI had longer lengths of ICU stay and hospital stay before pneumonia onset (median days, 3(0,8) vs. 0(0,2); 7(1,17.5) vs. 2(1,7), both *p* < 0.01) and more nosocomial pneumonia (86.7% vs. 54.7%, *p* < 0.01).

In comparison with KP pneumonia alone, KP pneumonia/KP-BSI was more severe, as evidenced by a higher APACHE II score (median, 24.5 vs. 17, *p* < 0.01), a higher SOFA score (median, 8.5 vs. 5, *p* < 0.01), more admission to the ICU (83.8% vs. 57.8%, *p* < 0.01) (Table 1).

### Biological indicators

The comparison of biological indicators between two groups is shown in Table 2 In comparison with the patients who had KP pneumonia alone, the patients with KP-pneumonia/KP-BSI had worse liver and kidney function, with higher values of albumin (ALB), total bilirubin (TBIL), aspartate aminotransferase (AST), alanine aminotransferase (ALT), and serum creatinine (SCr) (median ALB g/L, 30 vs. 32; TBIL μmol/L, 20 vs. 15; AST U/L, 46 vs. 34; ALT U/L, 46 vs. 30; SCr μmol/L, 73 vs. 63, all *p* < 0.01). Lower levels of hemoglobin (HB) and platelets (PLT) (median HB, 82 vs. 105 g/L; PLT, 138 vs. 169 × 10^9^/L, both *p* < 0.01) were observed in the patients with KP-pneumonia/KP-BSI, while white blood cell count (WBC) and absolute neutrophil count (ANC) were not significantly different. The inflammatory indexes of serum procalcitonin (PCT) and C-reactive protein (CRP) levels were significantly higher in the patients with KP-pneumonia/KP-BSI than in the patients with KP pneumonia alone.

**Table 2:** Comparison of biological indicators between groups of KP pneumonia alone and KP-pneumonia/KP-BSI

Biological indicators	Total (*n* = 409)	KP pneumonia alone (*n* = 274)	KP-pneumonia/KP-BSI (*n* = 135)	*P*-value
Liver and kidney function				
ALB (g/L) mean ± S.D.)	31 (28.9,35)	32 (29,35)	30 (27,34)	*P* < 0.01
ALT (U/L)	34 (23,61)	30 (20,49.5)	46 (31,74)	*P* < 0.01
AST (U/L)	38 (25,57.5)	34 (22,51)	46 (30,77)	*P* < 0.01
TBIL (umol/L)	16 (11,25)	15 (11,22.9)	20 (11,35)	*P* < 0.01
SCr (umol/L)	66 (50,95.5)	63 (50,85)	73 (51,124)	*P* < 0.01
Blood routine test				
WBC (× 10^9^/L) (IQR)	10.7 (7.5,14.1)	10.55 (7.9,14)	11.7 (5.9,15.9)	*P* = 0.88
ANC (IQR)	9 (6,12.6)	8.8 (6.2,12.2)	9.8 (4.7,13.9)	*P* = 0.70
HB(g/L)(IQR)	98 (78,117)	105 (87,126)	82 (71,97)	*P* < 0.01
PLT (× 109/L) (IQR)	163 (109,224.5)	169 (123,226)	138 (58,211)	*P* < 0.01
PCT (ng/ml) (IQR)	1.1 (0.3,3.6)	0.59 (0.2,1.68)	3.69 (1,21)	*P* < 0.01
CRP (mg/L)(IQR)	122 (58,188)	85.5 (49,161.7)	170 (85,241)	*P* < 0.01

*Abbreviations*: *KP* Klebsiella pneumoniae, *KP pneumonia /KP-BSI* KP pneumonia secondary Klebsiella pneumoniae bloodstream infections, *SD* standard deviation, *IQR* Interquartile range, *ALB* Albumin, *ALT* alanine aminotransferase, *AST* aspartate aminotransferase, *TBIL* Total bilirubin, *SCr* Serum creatinine, *WBC* White blood count, *ANC* Absolute neutrophil count, *HB* Hemoglobin, *PLT* Platelet, *PCT* Procalcitonin, *CRP* C-reactive protein

### Independent risk factors for KP-pneumonia/KP-BSI

**Fig. 2:**
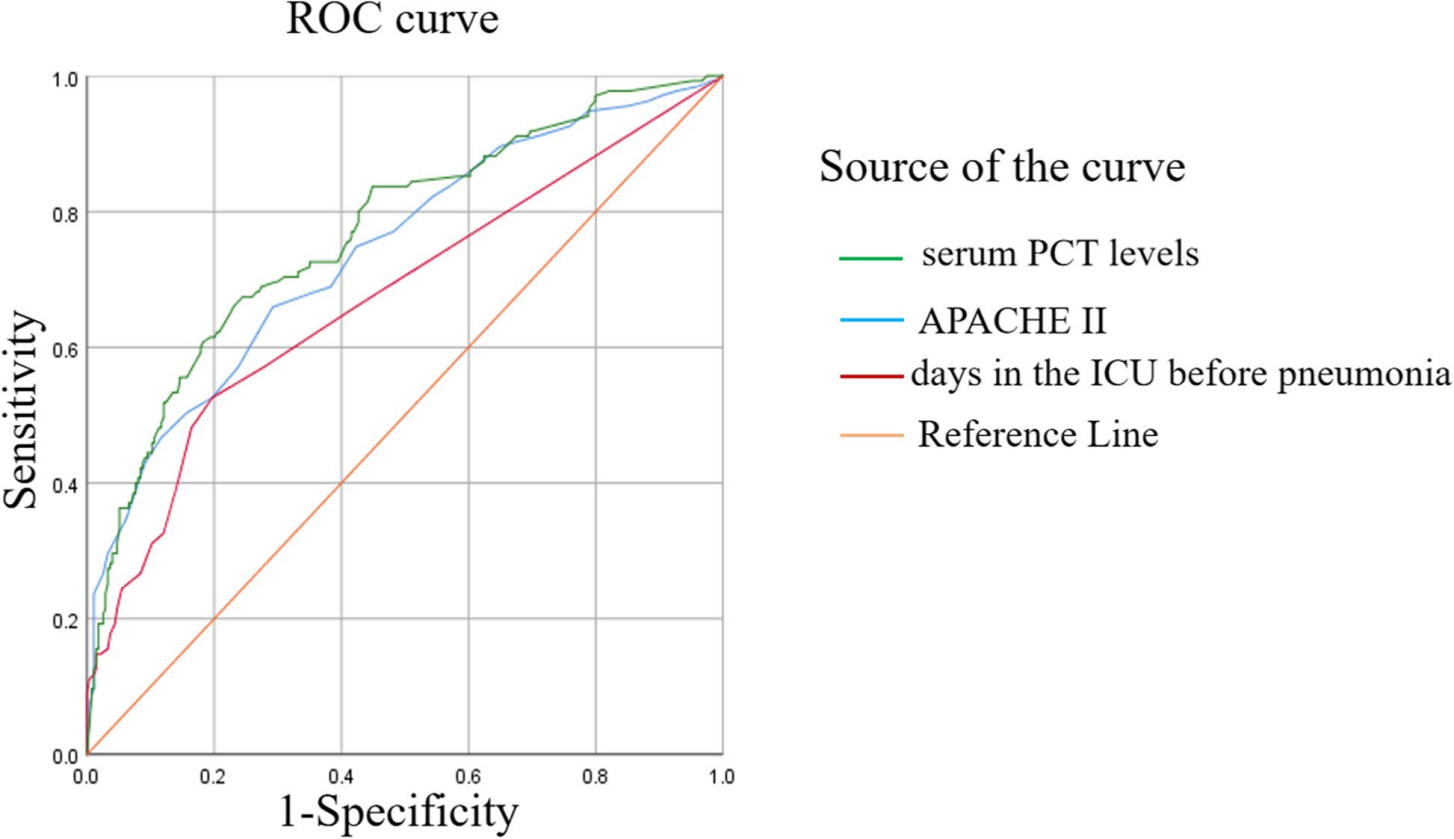
ROC curve analysis of serum PCT levels, APACHE II score and days in the ICU before pneumonia. Abbreviations: ROC, receiver operating characteristic; PCT, procalcitonin

To measure the optimal cutoff and calculate the individual specificity and sensitivity of APACHE II, serum PCT levels and days in the ICU before pneumonia, an ROC curve was plotted (Fig. 2). The AUC was 0.742 [95% confidence interval (CI), 0.689–0.794], 0.768 (95% CI, 0.718–0.818) and 0.674 (95% CI, 0.616–0.732) for APACHE II, serum PCT levels and the ICU days before pneumonia onset, respectively (Table 3). To combine the different parameters, the point with the smallest distance on the ROC curve was considered the optimal threshold. Based on the above data, the critical point of 21 was associated with a sensitivity of 65% and a specificity of 71% for APACHE II, 1.8 ng/ml for the serum PCT levels with a 66% sensitivity and a 77% specificity, and 2.5 days of ICU stay before pneumonia with a 53% sensitivity and an 80% specificity (Table 3).

**Table 3:** Results of the receiver operating characteristic curve analysis

Parameter	AUC (95%CI)	Cut-off	Sensitivity (%)	Specificity (%)
Serum PCT levels	0.768 (0.718–0.818)	1.8 ng/ml	66%	77%
APACHE II	0.742 (0.689–0.794)	21	65%	71%
Days in the ICU before pneumonia	0.674 (0.616–0.732)	2.5 days	53%	80%

*Abbreviations*: *PCT* procalcitonin, *CI* confidence interval, *AUC* area under the receiver operating characteristic curve

According to Table 4, the multiple logistic regression analysis showed that the independent risk factors for KP-pneumonia/KP-BSI were male sex (adjusted odds ratio [aOR] 3.7; 95% CI, 1.44–9.5), immunosuppression (aOR, 13.52; 95% CI, 2.53, 72.22), an APACHE II score higher than 21 (aOR, 3.39; 95% CI, 1.41–8.12), serum PCT value above 1.8 ng/ml (aOR, 6.37; 95% CI, 2.67–15.27), ICU stay of more than 2.5 days before pneumonia onset (aOR, 1.09; 95% CI, 1.02, 1.17), mechanical ventilation (aOR, 4.96; 95% CI, 1.2, 20.5), ESBL-positive KP (aOR, 12.93; 95% CI, 5.26–31.76), and inappropriate antibacterial therapy (aOR, 12.38; 95% CI, 5.36–28.58).

**Table 4:** Multivariable logistic regression of factors associated with KP-pneumonia/KP-BSI

Variable	Unadjusted OR (95%CI)	*P*-value	Adjusted OR (95%CI)	*P*-value
Male sex (%)	1.69 (1.03,2.75)	0.040	3.7 (1.44–9.5)	0.006
Hospitalization ward at KP pneumonia onset	3.78 (2.26,6.33)	0.010		
Chronic cardiac insufficiency	1.97 (1.13,3.43)	0.017		
Chronic renal failure	3.03 (1.13,8.14)	0.028		
Immunosuppressive	2.565 (1.08,6.10)	0.033	13.52 (2.53,72.22)	0.002
CRRT	4.86 (2.59,9.11)	0.000		
Mechanical ventilation	6.49 (3.34,12.61)	0.000	4.96 ( 1.2,20.5)	0.027
Blood transfusion	5.85 (3.53,9.70)	0.000		
Parenteral nutrition	1.77 (1.08,2.88)	0.023		
central line catheter	4.26 (2.36,7.69)	0.000		
Indwelling urinary catheter	4.27 (2.12,8.59)	0.00		
Prior hospitalized^a^	2.08 (1.36,3.18)	0.001		
Prior mechanical ventilation^a^	2.94 (1.73,5.00)	0.000		
Prior antibiotic exposure^a^	1.95 (1.27,2.98)	0.002		
Nosocomial infection	5.46 (3.15,9.47)	0.000		
ESBL-KP	13.89 (8.09,23.86)	0.000	12.93 (5.26–31.76)	0.000
Inappropriate antimicrobial therapy	5.96 (3.76,9.46)	0.000	12.38 (5.36–28.58)	0.000
Days of antibiotic use^b^ > 2.5d	4.86 (3.13,7.56)	0.000		
Days of hospitalization^b^ > 3.5d	3.49 (2.26,5.39)	0.000		
Days in ICU^b^ > 2.5d	1.13 (1.07,1.18)	0.000	1.09 (1.02,1.17)	0.013
APACHE II score > 21	4.69 (3.02,7.29)	0.000	3.39 (1.41–8.12)	0.006
SOFA score > 6.5	1.25 (1.18,1.32)	0.000		
PCT > 1.8 ng/mL	6.48 (4.12,10.2)	0.000	6.37 (2.67,5.23)	0.000
CRP > 162.5 mg/L	3.75 (2.42,5.8)	0.000		

*Abbreviations*: *KP* Klebsiella pneumoniae, *KP pneumonia /KP-BSI* KP pneumonia secondary Klebsiella pneumoniae bloodstream infections, *OR* odds ratio, *CI* confidence interval, *CRRT* Continuous renal replacement therapy, *ESBL-positive KP* klebsiella pneumoniae isolates producing extended spectrum β-lactamase, *ICU* Intensive care unit, *APACHE* Acute physiology and chronic health evaluation, *SOFA* Sequential organ failure assessment, *ALB* Albumin, *ALT* alanine aminotransferase, *AST* aspartate aminotransferase, *TBIL* Total bilirubin, *SCr* Serum creatinine, *WBC* White blood count, *ANC* Absolute neutrophil count, *HB* Hemoglobin, *PCT* Procalcitonin, *CRP* C-reactive protein

^a^in recent 3 months

^b^before sputum culture positive

### Antibiotic resistance and appropriate treatment

Compared to KP pneumonia alone, nosocomial acquired KP pneumonia in KP pneumonia/KP BSI was more common (86.7% vs. 54.7%, *p* < 0.01), and the rate of ESBL-positive K. pneumoniae was also higher (85.2% vs. 29.6%, *p* < 0.01), as evidenced by a lower ratio of bacterial susceptibility to Levofloxacin, Meropenem or Tigecycline (25.2% vs. 77.7%, 29.3% vs. 78.2%, and 14.885.2% vs. 94.9%, respectively, all *p* < 0.01) (Table 5). Regarding appropriate antibacterial therapy, there was also a significant difference between the groups of KP pneumonia/KP BSI and KP pneumonia (44.9% vs. 82.5%, *p* < 0.01).

**Table 5:** Antimicrobial susceptibility of KP and antimicrobial therapy in patients with KP pneumonia alone and KP-pneumonia/KP-BSI

Bacteriology	Total (*n* = 409)	KP pneumonia alone (*n* = 274)	KP-pneumonia/KP-BSI (*n* = 135)	*P*-value
Antimicrobial susceptibility,* n* (%)				
Amoxicillin-clavulanic acid	223 (54.5%)	203 (74.1%)	20 (14.8%)	*P* < 0.01
Amikacin	315 (77%)	244 (89.1%)	71 (52.6%)	*P* < 0.01
Aztreonam	221 (54%)	198 (82.3%)	23 (17%)	*P* < 0.01
Ceftazidime	232 (56.7%)	208 (76%)	24 (17.8%)	*P* < 0.01
Ciprofloxacin	246 (60.1%)	208 (75.9%)	38 (28.1%)	*P* < 0.01
Cefatriaxone	206 (49.6%)	189 (69%)	17 (12.6%)	*P* < 0.01
Cefoperazone -Sulbactam	237 (57.9%)	211 (77%)	23 (17%)	*P* < 0.01
Cefepime	242 (59.2%)	214 (78.1%)	28 (20.7%)	*P* < 0.01
Cefoxitin	233 (57%)	207 (75.5%)	26 (19.3%)	*P* < 0.01
Piperacillin-tazobactam	237 (57.9%)	208 (75.9%)	26 (19.2%)	*P* < 0.01
Levofloxacin	252 (61.6%)	213 (77.7%)	34 (25.2%)	*P* < 0.01
Imipenem	238 (58.2%)	211 (77%)	27 (20%)	*P* < 0.01
Meropenem	240 (58.7%)	214 (78.2%)	26 (29.3%)	*P* < 0.01
Tigecycline	375 (91.7%)	260 (94.9%)	115 (85.2%)	*P* < 0.01
Compound sulfamethoxazole	113 (27.6%)	42 (15.3%)	71 (52.6%)	*P* < 0.01
Appropriate antimicrobial therapy, *n* (%)	288 (70.1%)	227 (82.8%)	61 (45.2%)	*P* < 0.01

*Abbreviations*: *KP* Klebsiella pneumoniae, *KP pneumonia /KP-BSI* KP pneumonia secondary Klebsiella pneumoniae bloodstream infections

### Outcomes

In comparison with KP pneumonia alone, the incidence of septic shock in the patients with KP pneumonia/KP BSI was nearly 3 times higher (64.4% vs. 20.1%, *p* < 0.01); the duration of mechanical ventilation, length of ICU stay, and length of total hospital stay were all longer in the group of patients with KP pneumonia/KB BSI (median days, 15 (4, 34) vs. 4 (0,11); 19 (6,41) vs. 6 (0,14); 34 (19,57) vs. 17 (11,26), all *p* < 0.01). There was an overall crude mortality rate of 38.6%, which was significantly higher in the patients with KP pneumonia/KP BSI than in those with KP pneumonia alone (61.5% vs. 27.4%, *p* < 0.01) (Table 6).

**Table 6:** Outcomes of patients with KP pneumonia alone and KP-pneumonia/KP-BSI

Parameter	Total (*n* = 409)	KP pneumonia alone (*n* = 274)	KP-pneumonia/KP-BSI(*n* = 135)	*P*-value
Days of Mechanical ventilation, (M) (IQR)	7 (0,18)	4 (0,11)	15 (4,34)	*P* < 0.01
Total ICU days, (M) (IQR)	9 (1,20)	6 (0,14)	19 (6,41)	*P* < 0.01
Total hospital days, (M) (IQR)	20 (13,35)	17 (11,26)	34 (19,57)	*P* < 0.01
Cause Septic shock (n, %)	142 (34.7%)	55 (20.1%)	87 (64.4%)	*P* < 0.01
In-hospital mortality, (n,%)	158 (38.6%)	75 (27.4%)	83 (61.5%)	*P* < 0.01

*Abbreviations*: *KP* Klebsiella pneumoniae*, KP pneumonia /KP-BSI* KP pneumonia secondary Klebsiella pneumoniae bloodstream infections, *M* median, *IQR* interquartile range, *ICU* intensive care unit

## Discussion

To date, this is the largest study on the clinical characteristics and outcomes of secondary KP-BSI from KP pneumonia in mainland China. Several main findings were obtained from the current study. First, KP-pneumonia/KP-BSI is not rare and accounted for almost one-third of all patients with KP pneumonia. Second, many factors were related to KP-pneumonia/KP-BSI (Tables 1 and 2), whereas factors including male sex, immunosuppression, APACHE II score higher than 21, serum PCT above 1.8 ng/ml, ICU stay of more than 2.5 days before pneumonia onset, mechanical ventilation, ESBL-positive KP, and inappropriate antibacterial therapy were independently related to the occurrence of KP-pneumonia/KP-BSI from KP pneumonia alone (Table 4). Of note, independent risks such as ESBL-positive KP and inappropriate antibacterial therapy might be preventable or interventionable. Finally, the outcomes of the patients with KP-pneumonia/KP-BSI were worse than those of the patients with KP-pneumonia/KP-BSI alone.

Previous studies have also explored the risk factors for KP-BSI [8, 16]. Meatherall et al. [8] found that elderly patients and men were associated with KP bacteremia in a large Canadian health region from 2000 to 2007, whereas several risk factors, including dialysis, chronic liver disease, cancer and solid-organ transplantation, were independently associated with KP-BSI patients. However, this study did not specifically describe the source of KP-BSI. Among patients with carbapenem-resistant KP (CRKP) BSIs between January 2017 and September 2019, Zhu [16] reported that several risk factors, including APACHE II score and thrombocytopenia, were independently related to the development of CRKP-BSI from CRKP pneumonia. In contrast to these previous reports, our study strictly focused on secondary KP-BSI originating from KP pneumonia alone rather than any other source. Moreover, we selected KP, including CRKP, as the causative pathogen, which allowed us to specifically identify the risk factors or independent predictors of KP-pneumonia/KP-BSI.

Immunosuppressive status is a key determinant of the infection risk [29], and many articles have also confirmed that BSI is the main cause of mortality for immunosuppressed people [30–33]. Critically ill patients might always experience an immunosuppressive state and have an increased risk of acute infection [34, 35]. The APACHE II score has been widely used in the clinic to evaluate and predict the severity of diseases [36]. Consistent with these previous studies [29], the immunocompromised patients with immunosuppression or higher APACHE II/SOFA scores showed a trend toward BSI based on the univariate and multivariate analyses in the current study. According to our study, we concluded that the severity of pneumonia may be significant in determining the development of KP-BSI from KP pneumonia.

PCT is a valuable marker of bacterial infections, including pneumonia, bloodstream infections, and severe sepsis/sepsis shock, in different clinical settings [37]. Previous studies have shown that PCT values > 2 ng/ml strongly indicate sepsis or a severe bacterial infection [38]. In our study, the cutoff of PCT at 1.8 ng/ml suggested the possibility of the patient having KP pneumonia with secondary BSI. We also found that ICU stay before pneumonia onset was related to KP-pneumonia/KP-BSI, which was also confirmed by the results of previous studies that showed an independent association between previous ICU hospitalization days and secondary BSI, suggesting that these patients were likely to receive more invasive operations and treatments [39–41]. Indeed, we observed pronounced increases in invasive treatments such as mechanical ventilation, central line catheterization, indwelling urinary catheters and blood transfusions in KP-pneumonia/KP-BSI patients in comparison with patients who had KP-pneumonia alone. In light of these findings, it may be necessary to reduce unnecessary interventions and shorten hospital stays, especially the length of ICU stay, to prevent the development of KP pneumonia/KP-BSI.

Compared to KP pneumonia alone, KP pneumonia/KP BSI was more frequently caused by ESBL-positive KP and had a higher frequency of inadequate empirical antimicrobial therapy. Over the past few decades, overuse and misuse of antibiotics have led to an increase in antibiotic resistance, which has become a major public health problem [42]. The World Health Organization (in 2017) has placed ESBL-positive KP on the list of the most threatening superbugs along with *Pseudomonas aeruginosa* and *Acinetobacter baumannii* [43]. The antimicrobial resistance of KP is mainly related to ESBL production [44]. Previous findings found that indiscriminate antibiotic application upregulates ESBL expression, which plays a hydrolytic role in β-lactam antibiotic resistance [45]. In the current study, only 45.2% of the patients (61/135) with KP-pneumonia/KP-BSI received effective empiric therapy, while early and appropriate antimicrobial treatment is critical to reduce mortality in patients with bacteremia [46–48]. Taken together, these risk factors are very instructive to shed light on how to distinguish and prevent KP pneumonia from KP pneumonia/KP BSI.

Patients with KP-pneumonia/KP-BSI have worse outcomes, as evidenced by a higher incidence of septic shock, longer duration of mechanical ventilation, and longer lengths of ICU stay and total hospital stay. The overall in-hospital crude mortality rate was higher once KP pneumonia progressed to KP pneumonia/KP BSI. Our study showed that underlying host conditions influence the outcome, which was evidenced by the fact that patients with KP-pneumonia/KP-BSI are more likely to have chronic underlying diseases and other indicators of severe conditions, such as increased APACHE II and SOFA scores. In addition, a worse outcome was also found to have a strong association with the severity of pneumonia infection in this study, particularly with patients requiring mechanical ventilation, which is consistent with Baruah et al.’s study [49]. The relevant finding in our study was that the infections in patients with KP-pneumonia/KP-BSI were more often caused by resistant bacteria and that these patients had a higher frequency of inadequate empirical antimicrobial therapy, while antibiotic resistance was also associated with high rates of mortality in clinical patients [50]. Accordingly, patients with KP-pneumonia/KP-BSI seem to have a more severe illness and a worse prognosis.

This study has some limitations. First, it is a retrospective study in which all data were obtained through a review of the electronic medical records system, which may result in some important information not being accurately available. Second, this study was conducted in a single center with a relatively small number of patients. In addition, our hospital has a high reputation in the field of trauma treatment, and a substantial number of trauma patients are included in the study, which may lead to selection bias. Therefore, the results of the present study may not be applicable to other settings. Third, some important confounding variables of KP pneumonia/KP-BSI may not be included and analyzed in the current study due to its inherent drawbacks as retrospective study. Thus, it is necessary to conduct randomized controlled multi-center studies with large sample sizes to further clarify the risk factors and clinical features of KP pneumonia/KP BSI developed from KP pneumonia.

## Conclusions

KP-pneumonia/KP-BSI is relatively common in patients with KP pneumonia. Risk factors, including male sex, immunosuppression, APACHE II score higher than 21, serum PCT above 1.8 ng/ml, ICU stay of more than 2.5 days before pneumonia onset, mechanical ventilation, ESBL-positive KP, and inappropriate antibacterial therapy, are independent for KP-pneumonia/KP-BSI. Once patients with KP pneumonia develop secondary KP-BSI, they have a worse prognosis. Taken together, how to rapidly identify and prevent the development of secondary KP-BSI from KP pneumonia alone in a timely manner is critical and merits further investigation.

## Data Availability

All data generated or analyzed during this study are included in this manuscript, and the database is available from the first author ( drchenjuan@sina.com) upon reasonable request.
